# Value of CEA level determination in gallbladder bile in the diagnosis of liver metastases secondary to colorectal adenocarcinoma

**DOI:** 10.1590/S1516-31802001000300005

**Published:** 2001-05-02

**Authors:** Rita Maria Aparecida Monteiro Moura, Délcio Matos, Mário Mello Galvão, Giuseppe D'Ippólito, Jacob Sjzenfeld, Lídia Maria Giuliano

**Keywords:** Carcinoembryonic antigen (CEA), Bile, Metastases, Liver, Adenocarcinoma, Antígeno cárcinoembriônico (CEA), Bile, Metástase, Fígado, Adenocarcinoma

## Abstract

**CONTEXT:**

The relevance of colorectal adenocarcinoma lies in its high incidence, with the liver being the organ most frequently affected by distant metastases. Liver metastases occur in 40 to 50% of patients with colorectal adenocarcinoma, accounting for approximately 80% of deaths in the first three postoperative years. Nevertheless, despite this, they are occasionally susceptible to curative treatment.

**OBJECTIVE:**

The present investigation focused on the relationship between the level of carcinoembryonic antigen (CEA) in gallbladder bile and the presence of liver metastases secondary to colorectal adenocarcinoma.

**DESIGN:**

Diagnostic test study.

**SETTING:**

Surgical Gastroenterology Discipline at the São Paulo Hospital, São Paulo, Brazil.

**SAMPLE:**

Forty-five patients with colorectal adenocarcinoma were studied, 30 without liver metastases (group I), and 15 with liver metastases (group II). Diagnosis of liver metastases was made through computed tomography, magnetic resonance imaging and computed tomography during arterial portography. Samples of peripheral blood, portal system blood, and gallbladder bile were collected from patients during the surgical procedure. A control group composed of 18 organ donors underwent the same material collection procedures. CEA level determination was made through fluoroimmunoassay.

**RESULTS:**

Mean CEA value in peripheral serum was 2.0 ng/ml (range: 0.7 to 3.8 ng/ml) in the control group, 11.4 ng/ml (range: 0.5 to 110.3 ng/ml) in group I, and 66.0 ng/ml (range: 2.1 to 670 ng/ml) in group II. In the portal system, serum mean values found were 1.9 ng/ml (range: 0.4 to 5.0 ng/ml) in the control group, 15.3 ng/ml (range: 0.8 to 133.3 ng/ml) in group I, and 70.8 ng/ml (range: 1.8 to 725 ng/ml) in group II. Mean values found in gallbladder bile were 4.1 ng/ml (range: 1.0 to 8.6 ng/ml) in the control group, 14.3 ng/ml (range: zero to 93.0 ng/ml) in group I, and 154.8 ng/ml (range: 14.0 to 534.7 ng/ml) in group II.

**CONCLUSIONS:**

The CEA level in gallbladder bile is elevated in patients with liver metastases. Determination of CEA both in peripheral serum and in gallbladder bile enabled patients with liver metastases to be distinguished from those without such lesions. The level of CEA in gallbladder bile, however, seems to lead to a more accurate diagnosis of liver metastases secondary to colorectal adenocarcinoma.

## RESUMO

**CONTEXTO:**

A importância do adenocarcinoma colorretal está na sua alta incidência, sendo o fígado o órgão mais freqüentemente acometido por metástases à distância. As metástases hepáticas ocorrem em 40 a 50% dos pacientes portadores de adenocarcinoma colorretal e são responsáveis por cerca de 80% das mortes nos três primeiros anos de pósoperatório; mas, apesar disso, eventualmente são passíveis de tratamento curativo.

**OBJETIVO:**

Verificar a correlação entre os valores de CEA na bile vesicular e a presença de metástases hepáticas por adenocarcinoma colorretal.

**TIPO DE ESTUDO:**

Estudo de teste diagnóstico.

**LOCAL:**

Disciplina de Gastroenterologia Cirúrgica do Hospital São Paulo, São Paulo, Brasil.

**AMOSTRA:**

45 pacientes portadores de adenocarcinoma colorretal, dos quais 30 foram classificados como não portadores de metástases hepáticas (grupo I) e 15 como portadores de metástases hepáticas (grupo II). O diagnóstico de metástases hepáticas foi feito por tomografia computadorizada, ressonância magnética e porto tomografia computadorizada. Durante a cirurgia, os pacientes foram submetidos à coleta de sangue periférico e bile da vesícula biliar. Um grupo controle composto por 18 pacientes doadores de órgãos foi submetido aos mesmos procedimentos de coleta de material. A dosagem de CEA foi feita por método imunofluorimétrico.

**RESULTADOS:**

A média dos valores de CEA obtidos no soro periférico foi 2,0 ng/ml (0,7 a 3,8 ng/ml) no grupo controle; 11,4 ng/ml (0,5 a 110,3 ng/ml) no grupo I e 66,0 ng/ml 8 ng/ml (2,1 a 670 ng/ml) no grupo II. No soro do sistema portal, a média dos valores obtidos foi 1,9 ng/ml (0,4 a 5,0 ng/ml) no grupo controle; 15,3 ng/ml (0,8 a 133,3 ng/ml) no grupo I e 70,8 ng/ml (1,8 a 725 ng/ml) no grupo II. Na bile, a média observada foi 4, 1 ng/ml (1,0 a 8,6 ng/ml); 14,3 ng/ml (zero a 93,0 ng/ml) e 154,8 ng/ml (14,0 a 534,7 ng/ml), respectivamente, para os três grupos.

**CONCLUSÕES:**

Os valores de CEA na bile estão elevados em pacientes portadores de metástases hepáticas. Através, tanto da dosagem de CEA no soro periférico, quanto na bile, foi possível diferenciar pacientes sem metástases hepáticas daqueles portadores de tais lesões. A dosagem de CEA na bile, no entanto, foi mais acurada no diagnóstico de metástases hepáticas por adenocarcinoma colorretal.

## INTRODUCTION

The relevance of colorectal adenocarcinoma lies in its high incidence, with the liver being the organ most frequently affected by distant metastases.^1–2^ Liver metastases occur in 40 to 50% of patients with colorectal adenocarcinoma, accounting for approximately 80% of deaths in the first three postoperative years. Nevertheless, despite this, they are occasionally susceptible to curative treatment.^3–4^ The relevance of studies that seek the early diagnosis of such lesions is obvious. Diagnosis is particularly based on imaging techniques which, despite relevant advances, still present limitations, particularly with regard to very small lesions.^5–6^ Tumor markers are a further method that can be used in the diagnosis of liver metastases; in the case of colorectal adenocarcinoma, carcinoembryonic antigen (CEA) is the marker most widely used.^7^ It is conventionally determined in serum and, although being the most sensitive and specific, it is rather limited in the diagnosis of the primary tumor.^8^ Despite its increased sensitivity in the presence of liver metastases, it remains limited in cases of initial lesions.^9^ Owing to such limitations, several authors have studied CEA levels in gallbladder bile, aiming to improve their sensitivity and specificity for the diagnosis of liver metastases at earlier stages.^10–19^ This study aimed to verify the correlation between CEA levels in gallbladder bile and the presence of liver metastases in patients with colorectal adenocarcinoma.

## METHODS

The procedures that follow were in accordance with the ethical standards of the committee responsible for human experimentation and with the Helsinki Declaration of 1975, as revised in 1983.

From December 1993 to February 1996, 45 patients hospitalized in the Surgical Gastroenterology Discipline ward with a diagnosis of colorectal adenocarcinoma were enrolled. Patients with associated diseases, such as cholelithiasis, obstruction of the biliary pathways, intestinal inflammatory diseases, chronic or acute liver diseases and pancreatitis, were not included. For the classification of patients with and without liver metastases, three preoperative parameters were used, based on imaging techniques (computer tomography [CT], magnetic resonance [IRM] and computed arterial portography^7^ [CTAP]) and intraoperative assessment. Thus, group I, which included 30 patients without liver metastases, and group II, which included 15 patients with liver metastases were created. Eighteen organ-donor patients were used as the control group. None had cholelithiasis, obstruction of the biliary pathways, cirrhosis, liver schistosomiasis or pancreatitis. With regard to gender, the control group was composed of 13 males and 5 females, with ages ranging from 19 to 66 years, mean age 35.6 years. In group I, 9 males and 21 females were studied, with ages ranging from 28 to 83 years, mean age 57.2 years; whereas in group II, 7 males and 8 females were studied, with ages ranging from 30 to 80 years, mean age 58.3 years (Table 1).

**Table 1:** Distribution of mean, maximum and minimum values and standard deviation of CEA levels in peripheral serum

	control	Group I	Group II
n	16	30	15
mean	2.0	11.4	66.0
standard deviation	0.9	24.6	168.8
minimum	0.7	0.5	2.1
maximum	3.8	110.3	670.0

As for Dukes classification,^20^ Group I was composed of 6 patients presenting Dukes A classification, 8 Dukes B, 12 Dukes C, and 4 were not classified as they underwent no tumor resection.

Forty-one patients were submitted to IMR and CTAP in addition to CT. The 4 patients who failed to undergo CTAP already presented liver metastases detected by CT and IMR. Imaging was always analyzed by two single examiners from the Imaging Diagnosis Department, who considered the scanning either positive or negative, according to the presence or absence of images suggesting liver metastases. The surgical inventory was made by the surgeon following the collection of both gallbladder bile and portal system blood. Following the macroscopic assessment of the liver, bimanual palpation was performed. Whenever the surgeon had any doubt, biopsy of the lesion was performed.

Peripheral venous blood was collected during anesthetic induction, by direct puncture of an upper limb vein. Ten ml was collected into a dry tube, which was centrifuged to separate serum.

At surgery, all patients were submitted to material collection soon after the abdominal cavity was opened, prior to the handling of the tumor or to the surgical inventory. In the control group, bile collection was performed before the liver was excised. Gallbladder bile was collected by puncture of the gallbladder fundus after a purse string suture using absorbable material.

All collected material was centrifuged and the separated serum was stored in a freezer at −20 °C, until level determination was performed. The CEA level determination in serum was performed by using the Delfia® method.

The Kruskal-Wallis test^21^ was used, separately, to compare every CEA level found in the peripheral serum, portal system serum and bile among the groups studied. The Friedman test^22^ was used to compare CEA levels in the peripheral serum, portal system serum and bile, between each other, within each group. Whenever a statistically significant difference among the groups was detected, the multiple comparisons test was applied to identify the difference. The level of significance of the tests applied was 5% (0.05) for rejection of the null hypothesis.

In order to determine the optimal normality limit value for CEA levels in bile, i.e. the value which distinguished the control group patients from those with colorectal adenocarcinoma, a ROC (receiver operating characteristic) curve was drawn.^23^ Likewise, in order to decide what the best cutoff point was for the CEA level in bile and for the CEA level in peripheral serum, allowing groups I and II to be distinguished, two further ROC curves were drawn. To compare the level of CEA in bile with the level of CEA in peripheral serum, the corresponding ROC curve areas were matched by using a non-parametric test, whose level of significance was 5% (a ≤ 0.05) for rejection of the null hypothesis.

## RESULTS

CEA levels obtained in peripheral serum were as follows: in group I patients, values ranged from 0.5 to 110.3 ng/ml (mean: 11.4 ng/ml; standard deviation: 24.6 ng/ml); in group II patients, values ranged from 2.1 to 670.0 ng/ml (mean: 66.0 ng/ml; standard deviation: 168.8 ng/ml) and in the control group patients, values ranged from 0.7 to 3.8 ng/ml (mean: 2.0 ng/ml; standard deviation: 0.9 ng/ml). No significant difference was found between values obtained in group I and the control group. However, such values were significantly lower than the ones obtained in group II (P= 0.0002) (Table 1).

CEA levels obtained in bile were as follows: in group I patients, values ranged from zero to 93.0 ng/ml (mean: 14.3 ng/ml; standard deviation: 18.0 ng/ml); in group II patients, values ranged from 14.0 to 534.7 ng/ml (mean: 154.8 ng/ml; standard deviation: 193.0 ng/ml) and in the control group patients, values ranged from 1.0 to 8.6 ng/ml (mean: 4.1 ng/ml; standard deviation: 2.0 ng/ml). No significant differences were found between values obtained in group I and the control group. However, such values were significantly lower than the ones found in group II (P= 0.00000006) (Table 2).

**Table 2:** Distribution of mean, maximum and minimum values and standard deviation of CEA levels in bile

	Control	group I	group II
n	18	30	15
Mean	4.1	14.3	154.8
Standard deviation	2.0	18.0	193.0
Minimum	1.0	0.0	14.0
Maximum	8.6	93.0	534.7

CEA levels found in bile were significantly higher than the ones found in peripheral serum in the three groups studied, with the following values found for groups I, II and the control: P = 0.033, P = 0.001 and P = 0.0001, respectively.

**Figure 1:**
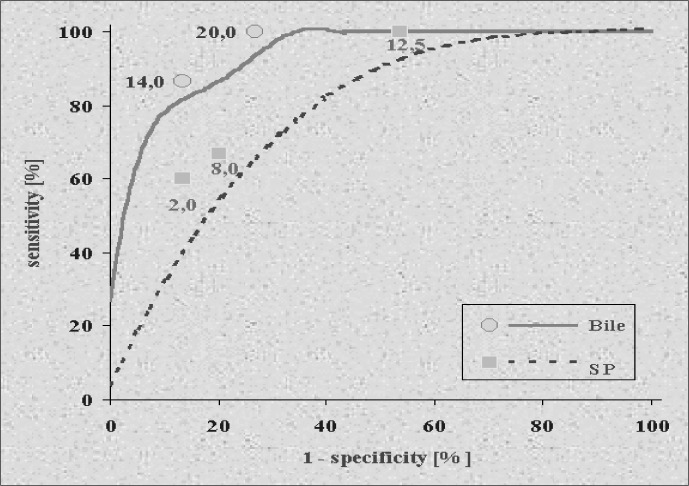
ROC curve to determine the level of CEA in bile (groups I and control).

The cutoff point for the CEA level in bile was 7.0 ng/ml, as this CEA level determined the largest area under the curve: 0.79 (Figure 1). Sensitivity found for this value was 63.3%, and specificity was 94%. Bile CEA levels tested were those close to the ones that presented optimal sensitivity and specificity in relation to the presence of colorectal adenocarcinoma in the 48 patients studied in groups I and control.

**Figure 2:**
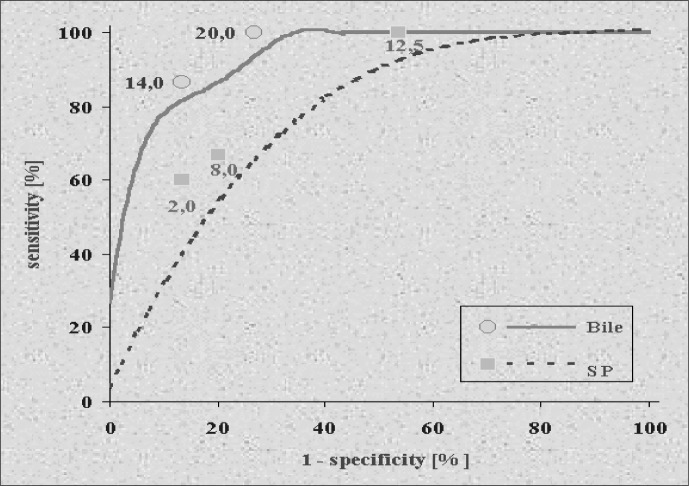
ROC curve to determine the level of CEA in bile and in peripheral serum.

Two CEA levels in bile determined the largest areas under the curve: 14.0 and 20.0 ng/ml, with an area of 0.87. For the 14.0 ng/ml limiting value, sensitivity found was 100% and specificity was 73.3. For the 20.0 ng/ml cutoff point, sensitivity found was 86.7% and specificity 86.7%. Three CEA values in peripheral serum presented the largest areas under the ROC curve: 2.0, 8.0 and 12.5 ng/ml, with an area of 0.73 (Figure 2). For the 2.0 ng/ml cutoff point, sensitivity found was 100% and specificity 46.7. For the 8.0 ng/ml cutoff point, sensitivity found was 66.7% and specificity 80.6%. For the 12.5 ng/ml cutoff point, sensitivity found was 60% and specificity 86.7%. Comparison between the areas of the ROC curves, drawn to determine the best cutoff points to determine CEA levels in bile and to determine CEA in the peripheral serum, showed a significant difference (P = 0.009) (Figure 2).

## DISCUSSION

CEA in peripheral serum was found to allow no distinction between patients in the control group from those without liver metastases, i.e. CEA in peripheral blood is not a good diagnosis test. This agrees with other studies in the literature^24–25^ that show CEA in peripheral serum to be of little sensitivity and specificity in cases of early colorectal adenocarcinoma. It was also found that CEA in peripheral serum allowed patients with liver metastases to be distinguished from those without liver metastases. This finding also agrees with data found in the literature^16–24^ showing improved sensitivity in the presence of liver metastases. CEA levels in bile showed the same statistical behavior as CEA in peripheral serum, i.e. they allowed no diagnosis of colorectal adenocarcinoma, but it was possible to distinguish patients with liver metastases from those without liver metastases. The distinction in this latter case had more efficacy, due to increased sensitivity and specificity. Statistical corroboration lies in the significant difference found between the ROC curves areas for both levels. And why is CEA in bile better able to detect liver metastases than CEA in peripheral serum? According to some authors,^17,19,21^ tumor cell products, with CEA among them, would be more concentrated in smaller amounts of bile than in larger amounts of serum and, furthermore, bile would be more exposed to such tumor products. These are plausible explanations, but there is controversy regarding the origin of CEA in bile, as CEA in bile may arise, at least in part, from the primary tumor.^26^ Experimental studies on the production, circulation, liver clearance and release of CEA in bile^27,28,29^ speak out against such possibility, although there are no studies on the release of CEA in bile in humans. A suggestion for studies that may solve such controversies, and which has already been presented by other authors,^23^ is to determine the level of CEA in bile prior to and following resection of the primary tumor.

A further issue which calls for discussion is that of false-positive results regarding CEA levels in bile, i.e. the patients without liver metastases who presented elevated CEA levels in bile. One of the possibilities is again the origin of CEA in the primary tumor, and a further possibility is cross-reactions. The existing methods are known to be adequate for determining CEA in serum, and failures may occur when they are used to determine CEA in bile.^10,11,16,18,30^ However, the most striking possibility for explaining elevated levels of CEA in bile in patients without detected liver metastases may relate to occult liver metastases.^31–32^ The only way to clear up such doubt is to follow up the patients without detected metastases who present high levels of CEA in bile, by performing serial tests to trace the appearance of such lesions. And why would it be important to know whether CEA in bile is predictive of the appearance of liver metastases? Because according to some authors,^10–12,14–16,18^ such a group of patients may benefit from some kind of prophylactic treatment to avoid the development of liver metastases.

## CONCLUSIONS

The main conclusion of this study is that CEA in bile increases in the presence of liver metastases secondary to colorectal adenocarcinoma. Second, it may be concluded that CEA in bile is better than CEA in peripheral serum for the diagnosis of liver metastases. Two further questions remain to be answered in subsequent studies. One deals with the origin of CEA in bile and the oher one concerns the false-positive results.

## References

[1] Boring CC, Squires TS, Tong T (1992). Cancer statistics 1992. CA Cancer J Clin.

[2] Festugato M, Alves MR, Schitt A, Spolavori A, Minotto C, Franke CA, Martini LC (1995). Câncer colorretal em Caxias do Sul. Rev. Cient AMECS.

[3] Gill PG, Morris PJ (1978). The survival of patients with colorectal cancer treated in a regional hospital. Br J Surg.

[4] Finlay IG, McArdle CS (1986). Occult hepatic metastases in colorectal carcinoma. Br J Surg.

[5] Ferrucci JT (1990). Liver tumor imaging: current concepts. AJR Am J Roentgenol.

[6] Moura RMAM, Matos D, D'Ippólito G, Triviño T, Sjzenfeld J, Matsumoto CA (1995). Estudo comparativo entre ressonância magnética e tomografia computadorizada na detecção de nódulos hepáticos em pacientes portadores de adenocarcinoma colorretal: resultados preliminares. Rev Bras Colo-proctol.

[7] Gold P, Freedman SO (1965). Demonstration of tumor-specific antigens in human colonic carcinoma by immunological tolerance and absorption techniques. J Exp Med.

[8] Fletcher RH (1986). Carcinoembryonic antigen. Ann Intern Med.

[9] Bell H (1982). Alpha-fetoprotein and carcinoembryonic antigen in patients with primary liver carcinoma, metastatic liver disease, and alcoholic liver disease. Scand J Gastroenterol.

[10] Yeatman TJ, Bland KI, Copeland EM (1989). Relationship between colorectal liver metastases and CEA levels in gallbladder bile. Ann Surg.

[11] Bromberg SH, Waisberg J, Pradal MG (1992). Níveis do antígeno carcinoembriônico na bile vesicular de pacientes com câncer colorretal: resultados preliminares. Rev Bras Colo-proctol.

[12] Ishida H, Hojo I, Gonda T (1993). Measurement of bile CEA levels in patients with colorectal cancer: is it of value for diagnosis of occult liver metastases aiming at prophylactic regional hepatic chemotherapy?. Gan To Kagaku Ryoho.

[13] Paul MA, Visser JJ, Cuesta MA, Meijer S (1993). Elevated biliary levels in colorectal patients: a prognostic factor?. Eur J Cancer.

[14] Paganuzzi M, Oneto M, Paoli M (1994). Carcinoembryonic antigen (CEA) in serum and bile of colorectal cancer patients with or without detectable liver metastases. Anticancer Res.

[15] Frikart L, Fournier K, Mach JP, Givel JC (1995). Potential value of biliary CEA assay in early detection of colorectal adenocarcinoma liver metastases. Eur J Surg Oncol.

[16] Paul MA, Visser JJ, Mulder C (1996). Detection of occult liver metastases by measurement of biliary carcinoembryonic antigen concentrations. Eur J Surg.

[17] Moura RMAM, Matos D, Rodrigues EBN, Giuliano LMP, Silberstein S, Abe O, Inokushi K, Takasaki K Relationship between colorectal liver metastases and CEA levels in gallbladder bile: preliminary results. World Congress of the International College of Surgeons 1996. 30, Kyoto, Japan, 25-29 November 1996.

[18] Novelli F, Trias M, Molina R, Filella X (1997). Detection of occult liver metastases in colorectal cancer by measurement of biliary carcinoembryonic antigen. Anticancer Res.

[19] Garcia BA, Madrona AP, Ayala MP, Paricio PP (1997). Utilidad dela determinación del antígeno carcinoembrionario en la bilis para la predicción del desarrollo de metástasis hepáticas tras la resección de cáncer colorectal. Med Clin Barcelona.

[20] Dukes CE, Bussey HJR (1958). The spread of rectal and its effect on prognosis. Br J Cancer.

[21] Siegel S, Siegel S (1975a). O caso de k amostras independentes. Estatística não paramétrica para as ciências do comportamento.

[22] Siegel S, Siegel S (1975b). O caso de k amostras relacionadas. Estatística não paramétrica para as ciências do comportamento.

[23] Fletcher RH, Fletcher SW, Wagner EH, Fletcher RH, Fletcher SW, Wagner EH (1996). Diagnóstico. Epidemiologia clínica: elementos essenciais.

[24] Manoukian N, Blum VF (1991). CEA nos tumores colorretais e gástricos. Gastroenterol Endosc Dig.

[25] Wang JY, Tang R, Chiang JM (1994). Value of carcinoembryonic antigen in the management of colorectal cancer. Dis Colon Rectum.

[26] Moody FG, Yeatman TJ, Bland KI, Copeland EM, Hollenbeck JE, Souba WW, Vogel SB, Kimura AK (1989). Discussion. Relationship between colorectal liver metastases and CEA levels in gallbladder bile. Ann Surg.

[27] Thomas P, Summers JW (1978). The biliary excretion of circulating asialo glycoproteins in the rat. Biochem Biophy Res Commun.

[28] Thomas P (1980). Studies on the mechanisms of biliary excretion of circulating glycoproteins. The carcinoembryonic antigen. Biochem J.

[29] Thomas P, Zamcheck N (1983). Role of the liver in clearance and excretion of circulating carcinoembryonic antigen (CEA). Dig Dis Sci.

[30] Scapa E, Haagensen DE, Cantarow W, Loewenstein MS, Thomas P, Zamcheck N (1985). Differences in CEA values determined by EIA and RIA in patients with benign and malignant biliary obstructions. Am J Clin Pathol.

[31] Finlay IG, McArdle CS (1982). The identification of patients at high risk following curative resection for colorectal carcinoma. Br J Surg.

[32] Goligher JC (1941). The operability of carcinoma of the rectum. Br Med J.

